# Synthesis, and spectroscopic and structural characterization of three new styryl­quinoline–benzimidazole hybrids

**DOI:** 10.1107/S2053229622010063

**Published:** 2022-10-25

**Authors:** Diana M. Ardila, Diego F. Rodríguez, Alirio Palma, Iván Díaz Costa, Justo Cobo, Christopher Glidewell

**Affiliations:** a Laboratorio de Síntesis Orgánica, Escuela de Química, Universidad Industrial de Santander, AA 678, Bucaramanga, Colombia; b Departamento de Química Inorgánica y Orgánica, Universidad de Jaén, 23071 Jaén, Spain; c School of Chemistry, University of St Andrews, Fife, KY16 9ST, United Kingdom; University of Strathclyde, United Kingdom

**Keywords:** synthesis, oxidative cyclo­condensation, styryl­quinoline, benzimidazole, mol­ecular hybrids, mol­ecular structure, mol­ecular conformation, hydrogen bonding, supra­molecular assembly, crystal structure

## Abstract

Three new 4-styryl­quinoline–benzimidazole hybrids have been synthesized using a reaction sequence in which 2-methyl­quinoline precursors first undergo selective oxidation by selenium dioxide to form the corresponding 2-formyl­quinoline inter­mediates, followed by oxidative cyclo­condensation reactions with benzene-1,2-diamine to yield the hybrid products. The formyl inter­mediates and the hybrid products have all been fully characterized using a combination of IR, ^1^H and ^13^C NMR spectroscopy, and high-resolution mass spectrometry, and the structures of the three hybrid products have been determined using single-crystal X-ray diffraction. Ethyl (*E*)-2-(1*H*-benzo[*d*]imidazol-2-yl)-4-(4-chloro­styr­yl)quinoline-3-carboxyl­ate, C_27_H_20_ClN_3_O_2_, (IIIa), and ethyl (*E*)-2-(1*H*-ben­zo[*d*]imidazol-2-yl)-4-(2-meth­oxy­styr­yl)quinoline-3-carboxyl­ate, C_28_H_23_N_3_O_3_, (IIIb), both crystallize in the solvent-free form with *Z*′ = 1, but ethyl (*E*)-2-(1*H*-benzo[*d*]imidazol-2-yl)-4-(4-methyl­styr­yl)quinoline-3-carboxyl­ate, C_28_H_23_N_3_O_2_, (IIIc), crystallizes as a partial hexane solvate with *Z*′ = 3, and the ester group in one of the independent mol­ecules is disordered over two sets of atomic sites having occupancies of 0.765 (7) and 0.235 (7). The mol­ecules of (IIIc) enclose continuous channels which are occupied by disordered solvent mol­ecules having partial occupancy. In all of the mol­ecules of (IIIa)–(IIIc), the styryl­quinoline fragment is markedly nonplanar. Different combinations of N—H⋯O and C—H⋯π hydrogen bonds generate supra­molecular assemblies which are two-dimensional in (IIIb) and (IIIc), but three-dimensional in (IIIa). Comparisons are made with the structures of some related com­pounds.

## Abstract 2

Three new 4-styryl­quinoline–benzimidazole hybrids have been synthesized in a two-step reaction sequence. The styryl­quinoline fragments are all nonplanar and the mol­ecules are linked into two- or three-dimensional arrays by N—H⋯O and C—H⋯π hydrogen bonds.

## Introduction

Among different privileged scaffolds, quinolines can be considered as one of the most versatile pharmacophores due to their presence in a wide variety of natural and synthetic mol­ecules. Quinoline derivatives exhibit a broad range of biological activity, such as anti­malarial (*e.g.* quinine and mefloquine) (Hu *et al.*, 2017 ▸; Kaur *et al.*, 2010 ▸; Orozco *et al.*, 2020 ▸), anti­viral (*e.g.* saquinavir) (Matada *et al.*, 2021 ▸), anti­cancer (*e.g.* camptothecin and topotecan) (Afzal *et al.*, 2015 ▸; Lauria *et al.*, 2021 ▸; Musiol, 2017 ▸; Yadav & Shah, 2021 ▸) and anti-­asthmatic (*e.g.* montelukast) (Matada *et al.*, 2021 ▸; Nayak, 2004 ▸). Quinoline derivatives are also frequently used as building blocks in the design and synthesis of new biologically active mol­ecular hybrids with the aim of developing new chemical entities for further clinical assays (Jagdale & Patil, 2019 ▸; Yadav & Shah, 2021 ▸).

The benzimidazole nucleus also constitutes a privileged scaffold which has been extensively studied as a potential building block for the development of biologically active mol­ecules with diverse applications as therapeutic agents, including anti­cancer agents (*e.g.* dovitinib and selumetinib) (Hernández-Romero *et al.*, 2021 ▸), anthelmintics (*e.g.* albendazole, mebendazole and thabendazole) (Salahuddin *et al.*, 2017 ▸) or antacids and anti-ulcer agents (*e.g.* omeprazole, lansoprazole and pantoprazole) (Gurvinder *et al.*, 2013 ▸).

Most of the synthetic methods for building the benzimidazole nucleus reported hitherto are based on cyclo­condensation reactions of benzene-1,2-di­amine (*o*-phenyl­enedi­amine) either with carboxaldehydes in the presence of Lewis acids or oxidizing agents (Agrawal *et al.*, 2012 ▸; Bellam *et al.*, 2017 ▸; Kidwai *et al.*, 2010 ▸; Lin & Yang, 2005 ▸; Singh *et al.*, 2000 ▸), or with carb­oxy­lic acids in strongly acidic conditions at high tem­per­atures (Cosimelli *et al.*, 2011 ▸; Singhal *et al.*, 2019 ▸). Because of the medicinal importance of quinoline and benz­imidazole derivatives, considerable efforts have been made in the development of novel quinoline–benzimidazole hybrids (Cosimelli *et al.*, 2011 ▸; Hranjec *et al.*, 2010 ▸; Mantu *et al.*, 2016 ▸; Perin *et al.*, 2016 ▸; Renhowe *et al.*, 2009 ▸; Yaragorla & Vijaya Babu, 2017 ▸). 
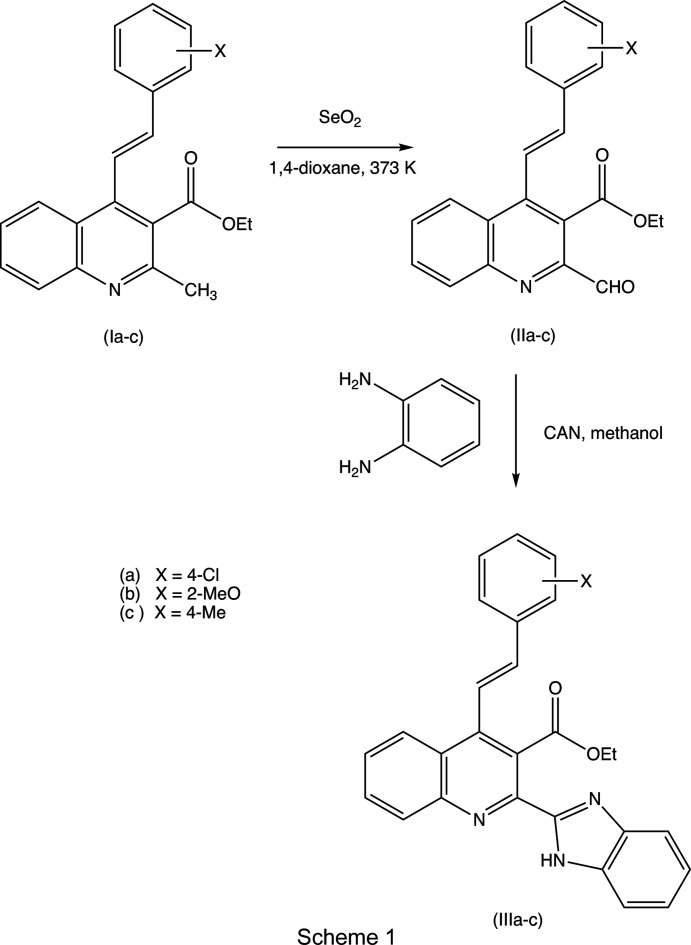


**Figure 1:**
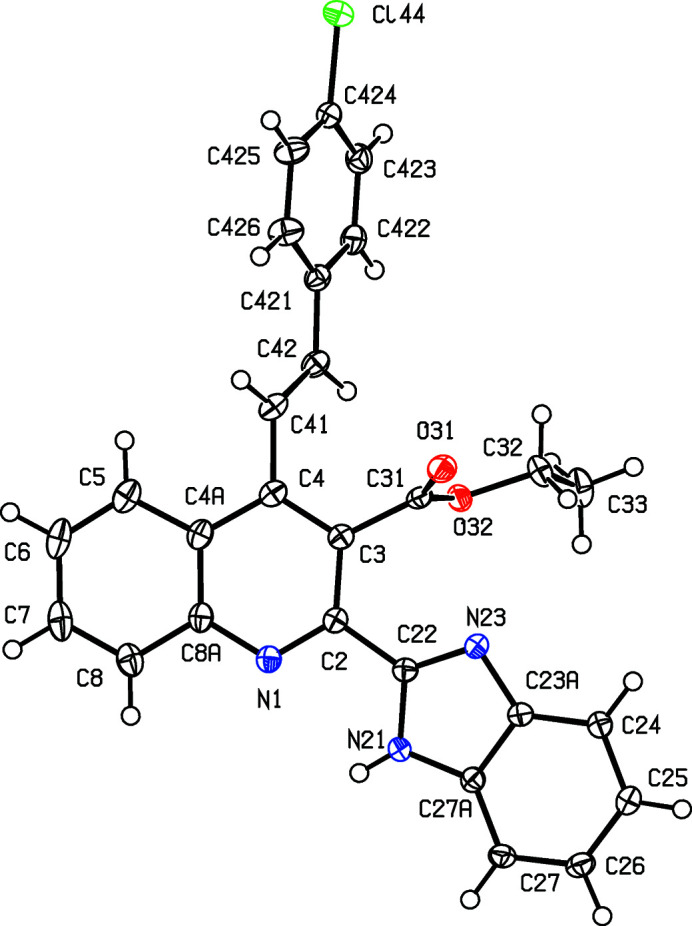
The mol­ecular structure of com­pound (IIIa), showing the atom-labelling scheme. Displacement ellipsoids are drawn at the 50% probability level.

With these considerations in mind, and as a continuation of our earlier work on the synthesis of polysusbstituted 4-sty­ryl­quinolines from 2′-amino­phenyl­chalcones and 1,3-dicarbonyl com­pounds (Meléndez *et al.*, 2020 ▸), we report here the syn­thesis and spectroscopic characterization of three representative examples of a novel class of mol­ecular hybrids of the type benzimidazole-4-styryl­quinoline, namely, ethyl (*E*)-2-(1*H*-benzo[*d*]imidazol-2-yl)-4-(4-chloro­styr­yl)quinoline-3-car­boxyl­ate, (IIIa), ethyl (*E*)-2-(1*H*-benzo[*d*]imidazol-2-yl)-4-(2-meth­oxy­styr­yl)quinoline-3-carboxyl­ate, (IIIb), and ethyl (*E*)-2-(1*H*-benzo[*d*]imidazol-2-yl)-4-(4-methyl­styr­yl)quinoline-3-carboxyl­ate, (IIIc), which differ only in the nature of the substituents in the benzene ring of the styryl fragment (see Scheme 1), along with the mol­ecular and supra­molecular structures of the hybrid products (IIIa)–(IIIc) (Figs. 1 ▸–3 ▸ ▸).

## Experimental

### Synthesis and crystallization

The 4-styryl­quinoline precursors of type (I) (see Scheme 1) were prepared using a previously reported method (Meléndez *et al.*, 2020 ▸; Rodríguez *et al.*, 2020 ▸). In the NMR data listed below, for compounds (III), unprimed ring atoms form part of the quinoline unit, ring atoms carrying a single prime form part of the benzimidazole unit and ring atoms carrying a double prime form part of the styryl unit (see Figs. 1 ▸ and 2 ▸).

For the synthesis of the formyl inter­mediates of type (II), a suspension of the appropriate 4-styryl­quinoline-3-carboxyl­ate (I) (Meléndez *et al.*, 2020 ▸; see Scheme 1) (1.0 mmol) and selenium dioxide (2.0 mmol) in 1,4-dioxane (5 ml) was stirred and heated at 373 K for the time required to com­plete the reaction. After the com­plete consumption of (I) [as monitored by thin-layer chromatography (TLC)], di­chloro­methane (15 ml) was added and the resulting suspension was filtered. The solvent was removed under reduced pressure and the resulting crude products were purified by flash column chromatography on silica gel using hexa­ne–ethyl acetate (10:1 *v*/*v*) as eluent to give the required formyl inter­mediates (IIa)–(IIc) as solid com­pounds.

Compound (IIa), ethyl (*E*)-4-(4-chloro­styr­yl)-2-formyl­quinoline-3-carboxyl­ate; yield 0.15 g (90%); m.p. 387–389 K; *R*_F_ = 0.28 (12.5% ethyl acetate–hexa­ne). FT–IR (ATR, cm^−1^): 1727 (C=O_form­yl_), 1706 (C=O_ester_), 1638 (C=N), 1612 (C=C_vin­yl_), 1558 (C=C_arom_), 1488 (C=C_arom_), 971 (=C—H_*trans*_). NMR (CDCl_3_): δ(^1^H) 10.19 (*s*, 1H, –COH), 8.28 (*dd*, *J* = 8.4, 1.3 Hz, 1H, H8), 8.20 (*dd*, *J* = 8.4, 1.5 Hz, 1H, H5), 7.88 (*ddd*, *J* = 8.4, 6.9, 1.5 Hz, 1H, H7), 7.74 (*ddd*, *J* = 8.3, 6.8, 1.3 Hz, 1H, H6), 7.51–7.48 (*m*, 2H, H2′, H6′), 7.45 (*d*, *J* = 16.5 Hz, 1H, H_A_C=), 7.41–7.38 (*m*, 2H, H3′, H5′), 7.03 (*d*, *J* = 16.5 Hz, 1H, =CH_B_), 4.45 (*q*, *J* = 7.2 Hz, 2H, –OCH_2_–), 1.33 (*t*, *J* = 7.2 Hz, 3H, –CH_3_); δ(^13^C) 192.5 (C=O_form­yl_), 167.4 (C=O_ester_), 148.7 (C2), 147.5 (C8a), 143.1 (C4), 138.0 (=CH_B_), 135.0 (C4′), 134.5 (C1′), 131.1 (C7), 131.0 (C8), 130.0 (C6), 129.2 (C3′, C5′), 128.2 (C2′, C6′), 127.5 (C4a), 125.4 (C5), 124.2 (C3), 121.3 (H_A_C=), 62.2 (–OCH_2_–), 14.1 (–CH_3_). HRMS (ESI^+^) *m*/*z* found for [*M* + H]^+^ 366.0892, C_21_H_16_ClNO_3_ requires 366.0891.

Compound (IIb), ethyl (*E*)-2-formyl-4-(2-meth­oxy­styr­yl)quinoline-3-carboxyl­ate; yield 0.16 g (97%); m.p. 372–373 K; *R*_F_ = 0.31 (12.5% ethyl acetate–hexa­ne). FT–IR (ATR, cm^−1^): 1726 (C=O), 1704 (C=O), 1597 (C=N), 1563 (C=C_vin­yl_), 1484 (C=C_arom_), 1462 (C=C_arom_), 970 (=C—H_*trans*_). NMR (CDCl_3_): δ(^1^H) 10.19 (*s*, 1H, –COH), 8.28 (*ddd*, *J* = 8.5, 1.4, 0.7 Hz, 1H, H5), 8.27 (*ddd*, *J* = 8.6, 1.4, 0.7 Hz, 1H, H8), 7.86 (*ddd*, *J* = 8.4, 6.9, 1.4 Hz, 1H, H7), 7.72 (*ddd*, *J* = 8.3, 6.9, 1.3 Hz, 1H, H6), 7.62 (*dd*, *J* = 7.6, 1.7 Hz, 1H, H6′), 7.54 (*d*, *J* = 16.7 Hz, 1H, H_A_C=), 7.42 (*d*, *J* = 16.7 Hz, 1H, =CH_B_), 7.34 (*ddd*, *J* = 8.3, 7.4, 1.7 Hz, 1H, H4′), 7.02 (*td*, *J* = 7.4, 1.1 Hz, 1H, H5′), 6.95 (*dd*, *J* = 8.3, 1.1 Hz, 1H, H3′), 4.47 (*q*, *J* = 7.2 Hz, 2H, –OCH_2_–), 3.88 (*s*, 3H, 2′-OCH_3_), 1.36 (*t*, *J* = 7.2 Hz, 3H, –CH_3_); δ(^13^C) 192.6 (C=O_formyl_), 167.3 (C=O_ester_), 157.5 (C2′), 148.6 (C2), 147.5 (C8a), 144.2 (C4), 134.7 (=CHB), 130.9 (C8), 130.8 (C7), 130.2 (C4′), 129.8 (C6), 127.7 (C4a), 127.5 (C6′), 125.7 (C5), 125.1 (C1′), 124.1 (C3), 121.2 (H_A_C=), 120.8 (C5′), 111.1 (C3′), 62.0 (–OCH_2_–), 55.5 (2′-OCH_3_), 14.0 (–CH_3_). HRMS (ESI^+^) *m*/*z* found for [*M* + H]^+^ 362.1388, C_22_H_19_NO_4_ requires 362.13868.

Compound (IIc), ethyl (*E*)-2-formyl-4-(4-methyl­styr­yl)quinoline-3-carboxyl­ate; yield 0.145 g (92%); m.p. 364–365 K; *R*_F_ = 0.32 (12.5% ethyl acetate–hexane). FT–IR (ATR, cm^−1^): 1707 (C=O_form­yl/ester_), 1628 (C=N), 1560 (C=C_vin­yl_), 1513 (C=C_arom_), 1463 (C=C_arom_), 987 (=C—H_*trans*_). NMR (CDCl_3_): δ(^1^H) 10.19 (*s*, 1H, –COH), 8.27 (*dd*, *J* = 8.3, 1.3 Hz, 1H, H8), 8.23 (*dd*, *J* = 8.4, 1.3 Hz, 1H, H5), 7.86 (*ddd*, *J* = 8.4, 6.9, 1.4 Hz, 1H, H7), 7.72 (*ddd*, *J* = 8.3, 6.9, 1.3 Hz, 1H, H6), 7.46 (*d*, *J* = 7.8 Hz, 2H, H2′, H6′), 7.43 (*d*, *J* = 16.6 Hz, 1H, H_A_C=), 7.23 (*d*, *J* = 7.8 Hz, 2H, H3′, H5′), 7.06 (*d*, *J* = 16.6 Hz, 1H, =CH_B_), 4.46 (*q*, *J* = 7.2 Hz, 2H, –OCH_2_–), 2.40 (*s*, 3H, 4′-CH_3_), 1.34 (*t*, *J* = 7.2 Hz, 3H, –CH_3_); δ(^13^C) 192.6 (C=O_form­yl_), 167.6 (C=O_ester_), 148.6 (C2), 147.5 (C8a), 143.7 (C4), 139.3 (=CH_B_, C4′), 133.3 (C1′), 131.0 (C7), 130.9 (C8), 129.8 (C6), 129.7 (C3′, C5′), 127.7 (C4a), 127.0 (C2′, C6′), 125.6 (C5), 124.2 (C3), 119.6 (H_A_C=), 62.1 (–OCH_2_–), 21.4 (4′-CH_3_), 14.3 (–CH_3_). HRMS (ESI^+^) *m*/*z* found for [*M* + H]^+^ 346.1452, C_22_H_19_NO_3_ requires 346.1438.

For the synthesis of the benzimidazole products of type (III), a suspension of the appropriate formyl derivatives (II) (1.0 mmol), *o*-phenyl­enedi­amine (1.0 mmol) and ceric am­monium nitrate (CAN) (10 mol%) in methanol (2 ml) was magnetically stirred at ambient tem­per­ature for the time required to com­plete the reaction. After the com­plete con­sumption of (II) (as monitored by TLC), methanol was removed under reduced pressure and the crude products were purified by flash column chromatography on silica gel using hexa­ne–ethyl acetate (8:1 *v*/*v*) as eluent to yield the target hybrid products (IIIa)–(IIIc), which were then recrystallized from hexa­ne–ethyl acetate (7:1 *v*/*v*), at ambient tem­per­ature and in the presence of air, to give yellow crystals suitable for single-crystal X-ray diffraction.

Compound (IIIa), ethyl (*E*)-2-(1*H*-benzo[*d*]imidazol-2-yl)-4-(4-chloro­styr­yl)quinoline-3-carboxyl­ate; yield 0.145 g (65%); m.p. 447–448 K; *R*_F_ = 0.31 (15% ethyl acetate–hexa­ne). FT–IR (ATR, cm^−1^): 3377 (N—H), 1716 (C=O), 1583 (C=N), 1488 (C=C_vin­yl_), 1455 (C=C_arom_), 1434 (C=C_arom_), 964 (=C—H_*trans*_). NMR (CDCl_3_): δ(^1^H) 10.69 (*s*, 1H, N—H), 8.15 (*ddd*, *J* = 8.4, 1.5, 0.7 Hz, 1H, H5), 8.13 (*ddd*, *J* = 8.4, 1.4, 0.7 Hz, 1H, H8), 7.82–7.79 (*m*, 1H, H4′), 7.78 (*ddd*, *J* = 8.4, 6.8, 1.4 Hz, 1H, H′), 7.61 (*ddd*, *J* = 8.3, 6.9, 1.3 Hz, 1H, H6), 7.53–7.51 (*m*, 1H, H′′), 7.51–7.49 (*m*, 2H, H6′′, H2′′), 7.49 (*d*, *J* = 16.6 Hz, 1H, H_A_C=), 7.41–7.38 (*m*, 2H, H3′′, H5′′), 7.33–7.29 (*m*, 1H, H6′), 7.27 (*ddd*, *J* = 8.5, 7.2, 1.0 Hz, 1H, H5′), 7.09 (*d*, *J* = 16.6 Hz, 1H, =CH_B_), 4.59 (*q*, *J* = 7.2 Hz, 2H, –OCH_2_–), 1.39 (*t*, *J* = 7.1 Hz, 3H, -CH_3_); δ(^13^C) 168.0 (C=O), 149.3 (C2), 147.2 (C8a), 144.6 (C3′a), 143.9 (C2′), 142.9 (C4), 137.7 (=CH_B_), 134.7 (C4′′), 133.6 (C1′′, C7′a), 130.8 (C7), 129.7 (C8), 129.1 (C3′′, C5′′), 128.2 (C2′′, C6′′), 127.9 (C6), 126.1 (C4a), 125.5 (C5), 125.4 (C3), 124.4 (C6′), 122.5 (C5′), 121.9 (H_A_C=), 121.0 (C4′), 111.1 (C7′), 62.2 (–OCH_2_–), 14.1 (–CH_3_). HRMS (ESI^+^) *m*/*z* found for [*M* + H]^+^ 454.1317, C_27_H_20_ClN_3_O_2_ requires 454.1317.

Compound (IIIb), ethyl (*E*)-2-(1*H*-benzo[*d*]imidazol-2-yl)-4-(2-meth­oxy­styr­yl)quinoline-3-carboxyl­ate; yield: 0.12 g (65%); m.p. 452–453 K, *R*_F_ = 0.30 (15% ethyl acetate–hexa­ne). FT–IR (ATR, cm^−1^): 3432 (N—H), 1713 (C=O), 1568 (C=N), 1487 (C=C_vin­yl_), 1467 (C=C_arom_), 1439 (C=C_arom_), 984 (=C—H_*trans*_). NMR (CDCl_3_): δ(^1^H) 10.72 (*s*, 1H, N—H), 8.25 (*ddd*, *J* = 8.5, 1.4, 0.6 Hz, 1H, H5), 8.12 (*ddd*, *J* = 8.6, 1.4, 0.6 Hz, 1H, H8), 7.82–7.80 (*m*, 1H, H4′), 7.77 (*ddd*, *J* = 8.3, 6.8, 1.4 Hz, 1H, H7), 7.66 (*dd*, *J* = 7.7, 1.7 Hz, 1H, H6′′), 7.60 (*ddd*, *J* = 8.3, 6.8, 1.3 Hz, 1H, H6), 7.58 (*d*, *J* = 16.7 Hz, 1H, H_A_C=), 7.52–7.50 (*m*, 1H, H7′), 7.50 (*d*, *J* = 16.7 Hz, 1H, =CH_B_), 7.35 (*ddd*, *J* = 8.3, 7.4, 1.7 Hz, 1H, H4′′), 7.32–7.24 (*m*, 2H, H5′, H6′), 7.03 (*td*, *J* = 7.5, 1.1 Hz, 1H, H5′′), 6.95 (*dd*, *J* = 8.3, 1.1 Hz, 1H, H3′′), 4.62 (*q*, *J* = 7.2 Hz, 2H, –OCH_2_–), 3.89 (*s*, 3H, 2′′-OCH_3_), 1.41 (*t*, *J* = 7.2 Hz, 3H, –CH_3_); δ(^13^C) 168.2 (C=O_ester_), 157.7 (C2′), 149.5 (C2), 147.2 (C8a), 144.7 (C3′a), 144.1 (C2′), 143.9 (C4), 134.3 (=CH_B_), 133.5 (C7′a), 130.6 (C7), 130.0 (C4′′), 129.6 (C8), 127.7 (C6), 127.4 (C6′′), 126.4 (C4a), 125.8 (C5), 125.4 (C1′′), 125.2 (C3), 124.2 (C6′), 122.3 (C5′), 121.8 (H_A_C=), 121.0 (C4′), 120.9 (C5′′), 111.1 (C7′), 62.0 (–OCH_2_–), 55.5 (2′′-OCH_3_), 14.0 (–CH_3_). HRMS (ESI^+^) *m*/*z* found for [*M* + H]^+^ 450.1815, C_28_H_23_N_3_O_3_ requires 450.1812.

Compound (IIIc), ethyl (*E*)-2-(1*H*-benzo[*d*]imidazol-2-yl)-4-(4-methyl­styr­yl)quinoline-3-carboxyl­ate; yield 0.135 g (65%); m.p. 418–419 K; *R*_F_ = 0.30 (15% ethyl acetate–hexa­ne). FT–IR (ATR, cm^−1^): 3439 (N—H), 1705 (C=O), 1564 (C=N), 1510 (C=C_vin­yl_), 1494 (C=C_arom_), 1434 (C=C_arom_), 976 (=C—H_*trans*_). NMR (CDCl_3_): δ(^1^H) 13.05 (*s*, 1H, N—H), 8.31 (*d*, *J* = 8.4 Hz, 1H, H5), 8.22 (*d*, *J* = 8.4 Hz, 1H, H8), 7.94 (*t*, *J* = 7.6 Hz, 1H, H7), 7.75 (*t*, *J* = 7.6 Hz, 1H, H6), 7.68–7.64 (*m*, 2H, H4′, H7′), 7.64 (*d*, *J* = 16.5 Hz, 1H, H_A_C=), 7.61–7.59 (*m*, 2H, H2′′, H6′′), 7.31–7.24 (*m*, 4H, H5′, H6′, H3′′, H5′′), 7.08 (*d*, *J* = 16.5 Hz, 1H, =CH_B_), 4.42 (*q*, *J* = 7.2 Hz, 2H, –OCH_2_–), 2.36 (*s*, 3H, 4′′-CH_3_), 1.27 (*t*, *J* = 7.1 Hz, 3H, –CH_3_); δ(^13^C) 167.6 (C=O), 149.7 (C2), 147.1 (C8a), 144.8 (C2′), 144.1 (C3′a), 143.5 (C4), 139.1 (C4′′), 138.5 (=CH_B_), 135.1 (C1′′), 133.7 (C7′a), 131.8 (C7), 129.9 (C3′′, C5′′), 129.7 (C8), 128.7 (C6), 127.6 (C2′′, C6′′), 126.3 (C5), 125.9 (C4a), 125.4 (C3), 124.3 (C6′), 122.6 (C5′), 120.9 (H_A_C=), 120.2 (C4′), 112.7 (C7′), 61.8 (–OCH_2_–), 21.4 (4′′-CH_3_), 14.4 (–CH_3_). HRMS (ESI^+^) *m*/*z* found for [*M* + H]^+^ 434.1860, C_28_H_23_N_3_O_2_ requires 434.1863.

### Refinement

Crystal data, data collection and refinement details for com­pounds (IIIa)–(IIIc) are summarized in Table 1 ▸. For each of these com­pounds, one bad outlier reflection, *i.e.* 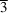 96 for (IIIa), 303 for (IIIb) and 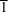 05 for (IIIc), was omitted from the data set. All H atoms were located in difference maps. H atoms bonded to C atoms were then treated as riding atoms in geometrically idealized positions, with C—H distances of 0.95 (alkenic and aromatic), 0.98 (CH_3_) or 0.99 Å (CH_2_) and with *U*_iso_(H) = *kU*_eq_(C), where *k* = 1.5 for the methyl groups, which were permitted to rotate but not to tilt, and 1.2 for all other H atoms. For the H atoms bonded to N atoms, the atomic coordinates were refined with *U*_iso_(H) = 1.2*U*_eq_(N), giving the N—H distances shown in Table 3.

For com­pound (IIIa), the final difference map contained one fairly large maximum, 0.61 e Å^−3^, at 0.5175, 0.8375, 0.7907. An attempt to treat this as the O atom of a partial-occupancy water mol­ecule gave a refined occupancy of 0.057 (3), but the angles subtended at this site by every pair of potential donors and/or acceptors which were within plausible hydrogen-bonding range were all less than 60°, some of them barely half the idealized tetra­hedral value. Accordingly, this possibility was discounted.

For com­pound (IIIc), the crystals were consistently of poor quality; this com­pound crystallizes in the space group *P*2_1_/*n* with *Z*′ = 3 and, for the best crystal examined, the *R*_int_ value was 0.176. In mol­ecule 1 of (IIIc), containing atom N11, the ester group is disordered over two sets of atomic sites having unequal occupancy. For the minor disorder com­ponent, the bonded distances and the [1,3] nonbonded distances were restrained to have the same values as the corresponding distances in the major com­ponent, subject to s.u. values of 0.01 and 0.02 Å, respectively. In addition, the anisotropic displacement parameters for pairs of partial-occupancy atoms within essentially the same physical space were constrained to be equal. Conventional refinement then converged only to *R*_1_ = 0.132 and *wR*_2_ = 0.391, and examination of the structure of (IIIc) at this point using *PLATON* (Spek, 2020 ▸) confirmed that no additional crystallographic symmetry was present and that twinning was also absent. However, *PLATON* showed that the structure formed by the mol­ecules of (IIIc) enclosed two voids, centred at (0,0,0) and ( 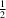 , 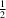 , 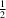 ) and each of volume *ca* 314 Å^3^, and that corresponding voids in unit cells related by translation along [010] are connected, thus forming continuous channels along (0, *y*, 0) and ( 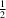 , *y*, 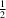 ). Further examination of this structure using the SQUEEZE procedure (Spek, 2015 ▸) indicated that each void contained around 55 electrons not hitherto accounted for, equivalent to just over one mol­ecule of hexane per void. The largest peaks in the difference map for (IIIc) lie within the channels, in the form of a zigzag chain, but no convincing solvent model could be developed from these peaks. It seems possible that the channels contain partial-occupancy disordered and possibly mobile hexane mol­ecules. Accordingly, the reflection data were subjected to the SQUEEZE procedure (Spek, 2015 ▸), and the resultant modified reflection file was used for the refinement reported here; the final refined values of the site-occupancy factors for the disordered ester group were 0.765 (7) and 0.235 (7).

## Results and discussion

The synthesis of the hybrid products (IIIa)–(IIIc) (see Scheme 1) starts from the precursor ethyl (*E*)-2-methyl-4-sty­ryl­quinoline-3-carboxyl­ates (Ia)–(Ic), using methods recently reported by us (Meléndez *et al.*, 2020 ▸). The conversion of precursors (Ia)–(Ic) to the formyl inter­mediates (IIa)–(IIc) was effected by selective oxidation of the 2-methyl group using selenium dioxide in refluxing 1,4-dioxane as the oxidant (Yaragorla & Vijaya Babu, 2017 ▸). The inter­mediates were isolated in yields of over 90%, and the conversion of the 2-methyl group to a 2-formyl group was confirmed by both the ^1^H and ^13^C NMR spectra (see Section 2.1) Finally, the formyl inter­mediates (IIa)–(IIc) were successfully converted into the target hybrid products (IIIa)–(IIIc) in yields of 65% by means of an oxidative cyclo­condensation reaction with *o*-phenyl­enedi­amine (1,2-di­amino­benzene), promoted by cerium(IV) ammonium nitrate (CAN) (see Scheme 1).

Compounds (IIa)–(IIc) and (IIIa)–(IIIc) were all fully characterized using IR, ^1^H and ^13^C NMR spectroscopy, and high-resolution mass spectrometry (see Section 2.1). The form­ation of the required benzimidazole–quinoline mol­ecular hybrid products (IIIa)–(IIIc) was confirmed by the disappearance of the formyl signals from both the ^1^H and ^13^C NMR spectra, and their replacement by new sets of signals corresponding to the five H atoms and seven C atoms of the newly formed benzimidazole ring, and by the appearance of new signals in the IR spectra corresponding to the N—H unit of the newly-formed benzimidazole ring.

The precursors of type (I) were prepared (Meléndez *et al.*, 2020 ▸; Rodríguez *et al.*, 2020 ▸) using a two-step reaction sequence starting from 2-amino­aceto­phenone, a substituted benzaldehyde and a 1,3-dicarbonyl com­pound. With such simple starting materials, a wide range of substituted derivatives is readily available, opening the way to the formation of a rich and diverse library of substituted styryl­quinoline–benzimidazole products and their analogues.

**Figure 3:**
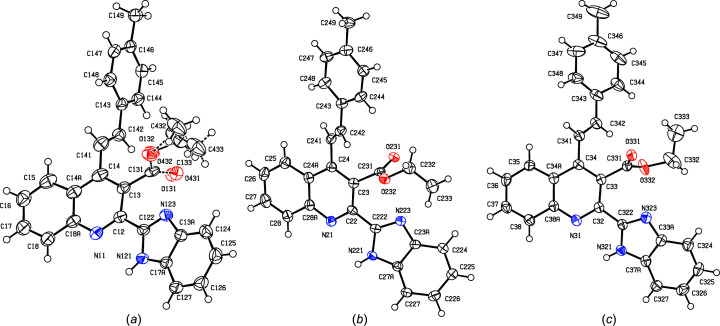
The three independent mol­ecules of com­pound (IIIc), showing the atom-labelling schemes for (*a*) mol­ecule 1, where the minor disorder com­ponent has been drawn using broken lines, (*b*) mol­ecule 2 and (*c*) mol­ecule 3. Displacement ellipsoids are drawn at the 50% probability level.

The constitutions of com­pounds (IIIa)–(IIIc), which were deduced from the spectroscopic data, were fully confirmed by the results of single-crystal X-ray diffraction (Figs. 1 ▸–3 ▸ ▸), which additionally provided information on the mol­ecular conformations and the inter­molecular inter­actions in the solid state. Compound (IIIc) crystallizes with *Z*′ = 3, but a search for possible additional crystallographic symmetry revealed none; it will be convenient to refer to the mol­ecules of (IIIc) containing atoms N11, N21 and N31 (Fig. 3 ▸) as mol­ecules 1–3, respectively.

The mol­ecules of com­pounds (IIIa)–(IIIc) exhibit no inter­nal symmetry, as indicated by the key torsion angles (Table 2 ▸). They are thus not superimposable upon their mirror images and hence they are conformationally chiral (Moss, 1996 ▸; Flack & Bernardinelli, 1999 ▸). In each com­pound, the styryl­quinoline fragment is nonplanar, as indicated by the values of the C3—C4—C41—C42 torsion angle (Table 2 ▸). We have noted previously (Vera *et al.*, 2022 ▸) that 4-styryl­quinoline derivatives typically have nonplanar skeletons, whereas 2-sty­ryl­quinolines and 8-styryl­quinolines typically have planar skeletons.

In all the mol­ecules of (IIIa)–(IIIc), the benzimidazole fragments have the N—H unit directed away from the ester group, so precluding the possibility intra­molecular N—H⋯O hydrogen bonding; the pyridine and imidazole rings are not coplanar, as shown by the dihedral angles between their planes (Table 2 ▸). In one of the mol­ecules of (IIIc), the ester group is disordered over two sets of atomic sites, having occupancies 0.765 (7) and 0.235 (7) [see Fig. 3 ▸(*a*)]. The only 2-benzimidazolyl­quinoline derivatives recorded in the Cam­bridge Structural Database (CSD; Groom *et al.*, 2016 ▸) are titanium com­plexes in which the quinolone N atom and one of the imidazole N atoms are both coordinated to Ti, forming a five-membered ring, and hence the conformations of the organic ligands in these com­pounds are not usefully com­parable with those in metal-free systems. The orientations of the ester groups relative to the pyridine ring may be a consequence of the N—H⋯O hydrogen bond (see below), as in every mol­ecule in (IIIa)–(IIIc), the carbonyl O atom acts as an acceptor in such an inter­action.

While com­pounds (IIIa) and (IIIb) crystallize in the sol­vent-free form, com­pound (IIIc) contains disordered solvent within continuous channels; hence, it is to be expected that the supra­molecular assembly for (IIIc) will differ from those of (IIIa) and (IIIb), as the *Z*′ value immediately indicates.

For com­pound (IIIa), the supra­molecular assembly is based upon three hydrogen bonds, one of the N—H⋯O type and two of the C—H⋯π type (Table 2 ▸), and the combination of these three inter­actions links the mol­ecules of (IIIa) into a three-dimensional framework structure. However, the for­mation of the framework is readily analysed in terms of three simple substructures (Ferguson *et al.*, 1998*a* ▸,*b* ▸; Gregson *et al.*, 2000 ▸), each involving just one type of hydrogen bond.

**Figure 4:**
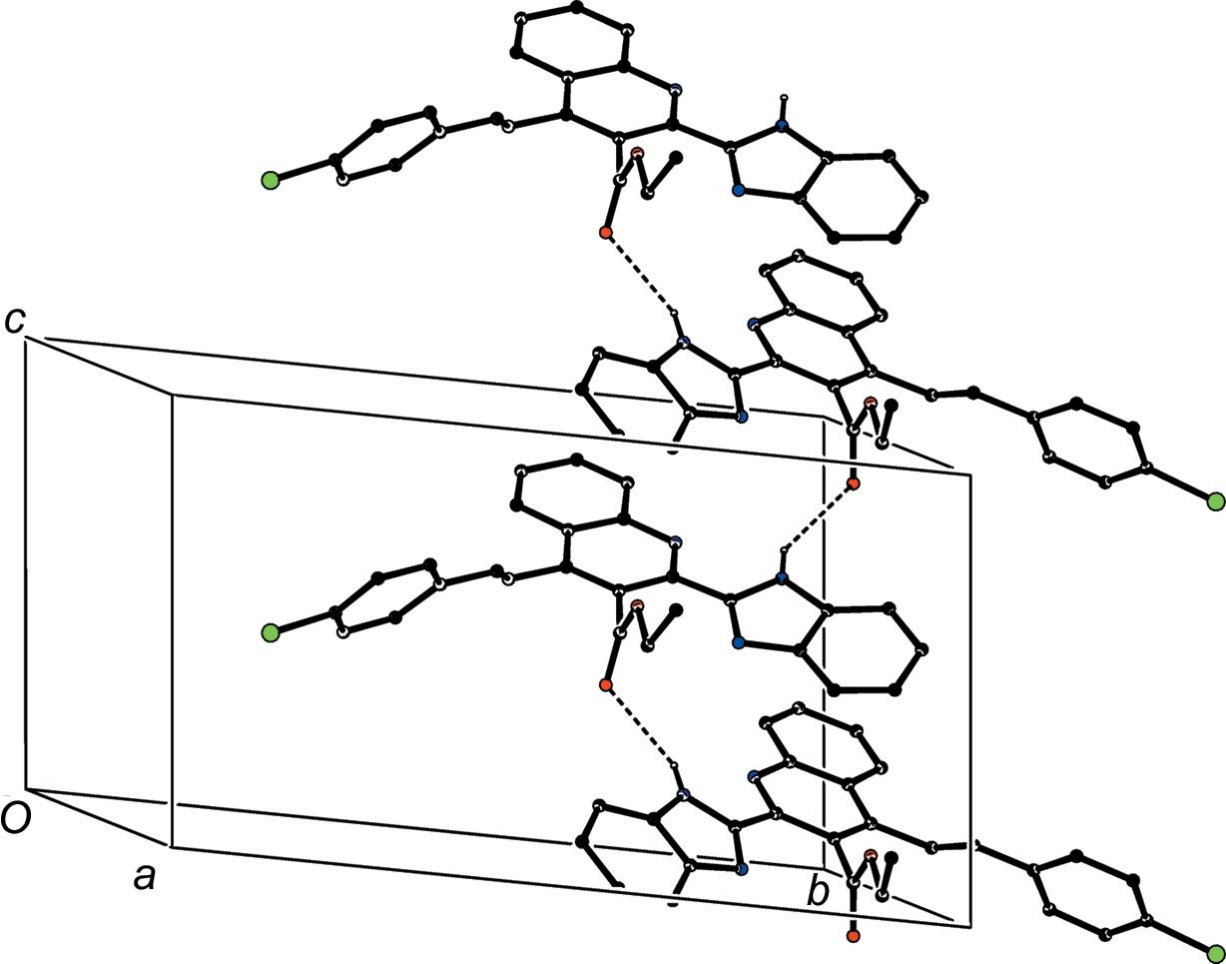
Part of the crystal structure of com­pound (IIIa), showing the formation of a *C*(7) chain built from N—H⋯O hydrogen bonds and running parallel to the [001] direction. Hydrogen bonds are drawn as dashed lines and, for the sake of clarity, H atoms not involved in the motif shown have been omitted.

**Figure 5:**
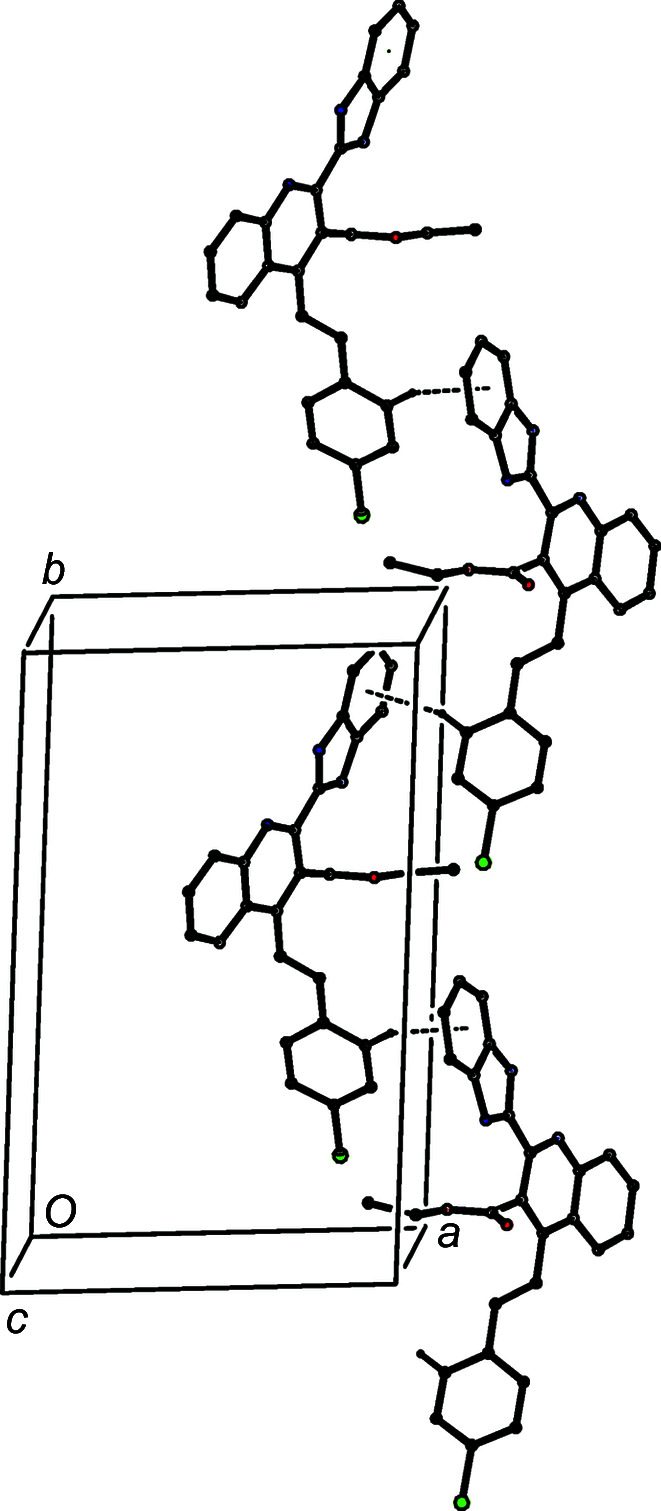
Part of the crystal structure of com­pound (IIIa), showing the formation of a chain built from C—H⋯π hydrogen bonds and running parallel to the [010] direction. Hydrogen bonds are drawn as dashed lines and, for the sake of clarity, H atoms not involved in the motif shown have been omitted.

**Figure 6:**
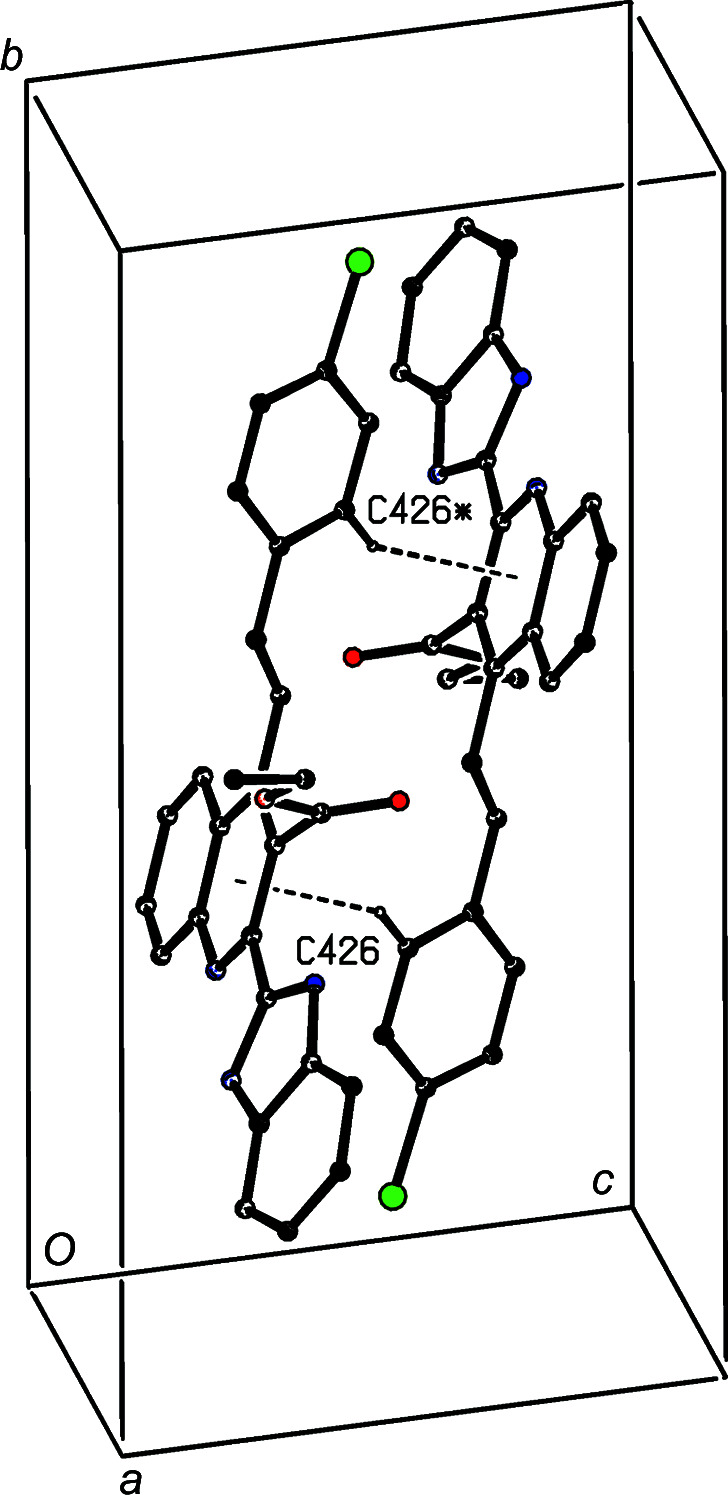
Part of the crystal structure of com­pound (IIIa), showing the formation of a cyclic centrosymmetric motif built from C—H⋯π hydrogen bonds and linking adjacent (100) sheets. Hydrogen bonds are drawn as dashed lines and, for the sake of clarity, H atoms not involved in the motif shown have been omitted. The atom marked with an asterisk (*) is at the symmetry position (−*x* + 1, −*y* + 1, −*z* + 1).

In the first substructure, mol­ecules of (IIIa) which are related by the *c*-glide plane at *y* = 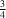 are linked by N—H⋯O to form a *C*(7) (Etter, 1990 ▸; Etter *et al.*, 1990 ▸; Bernstein *et al.*, 1995 ▸) chain running parallel to the [001] direction (Fig. 4 ▸). A second substructure involves the C—H⋯π hydrogen bond having atom C422 as the donor (Table 2 ▸); this inter­action links mol­ecules of (IIIa) which are related by the 2_1_ screw axis along (1, *y*, 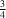 ) to form a chain running parallel to the [010] direction (Fig. 5 ▸). The combination of the chains along [010] and [001] gives rise to a sheet lying parallel to (100). Adjacent sheets are then linked by the third substructure, which is built from C—H⋯π hydrogen bonds having atom C426 as the donor, which links inversion-related mol­ecules from adjacent sheets (Fig. 6 ▸), so com­pleting the three-dimensional assembly.

**Figure 7:**
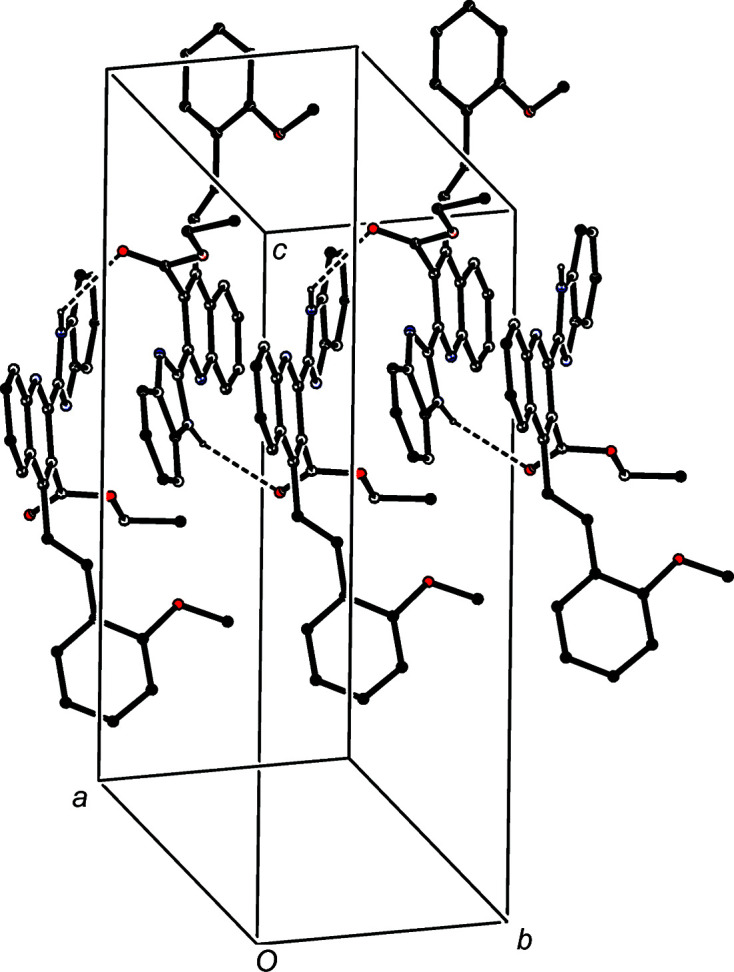
Part of the crystal structure of com­pound (IIIb), showing the formation of a *C*(7) chain built from N—H⋯O hydrogen bonds and running parallel to the [010] direction. Hydrogen bonds are drawn as dashed lines and, for the sake of clarity, H atoms not involved in the motif shown have been omitted.

**Figure 8:**
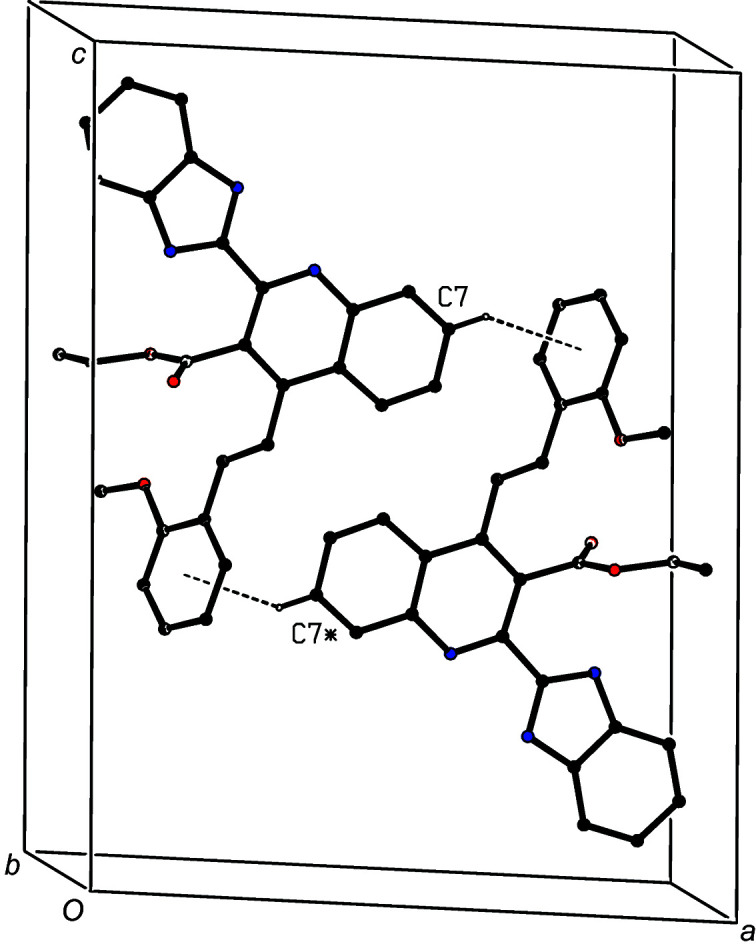
Part of the crystal structure of com­pound (IIIb), showing the formation of a cyclic centrosymmetric motif built from C—H⋯π hydrogen bonds and linking adjacent [010] chains. Hydrogen bonds are drawn as dashed lines and, for the sake of clarity, H atoms not involved in the motif shown have been omitted. The atom marked with an asterisk (*) is at the symmetry position (−*x* + 1, −*y* + 1, −*z* + 1).

An N—H⋯O hydrogen bond is also present in the structure of com­pound (IIIb) (Table 3 ▸), and this links mol­ecules which are related by the 2_1_ screw axis along ( 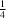 , y, 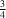 ) to form a *C*(7) chain running parallel to the [010] direction (Fig. 7 ▸). The C—H⋯π hydrogen bond (Table 3 ▸) links inversion-related mol­ecules in adjacent chains into a cyclic centrosymmetric motif (Fig. 8 ▸), which links the [010] chains into a sheet lying parallel to (101). There are no direction-specific inter­actions between adjacent sheets in (IIIb); the only other short inter­molecular contact in the structure involves a C—H bond in a methyl group, which is almost certainly undergoing rapid rotation about the adjacent C—C bond (Riddell & Rogerson, 1996 ▸, 1997 ▸).

**Figure 9:**
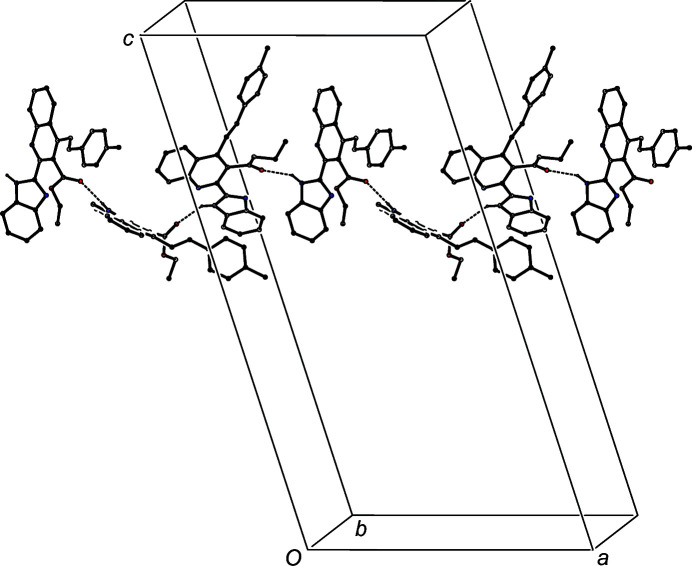
Part of the crystal structure of com­pound (IIIc), showing the formation of a 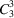 (21) chain built from N—H⋯O hydrogen bonds and running parallel to the [100] direction. Hydrogen bonds are drawn as dashed lines and, for the sake of clarity, the minor disorder com­ponent and the H atoms not involved in the motif shown have been omitted.

The hydrogen-bonded supra­molecular assembly in com­pound (IIIc), where *Z*′ = 3, is also two-dimensional and can readily be analysed in terms of two simple substructures. In the first of these, the three independent N—H⋯O hydrogen bonds of com­pound (IIIc) are linked by two N—H⋯O hydrogen bonds (Table 3 ▸) to form a linear three-mol­ecule aggregate, and aggregates of this type which are related by translation are linked by a third N—H⋯O hydrogen bond to form a 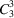 (21) chain running parallel to the [100] direction (Fig. 9 ▸). The formation of this chain is thus analogous to those formed in com­pounds (IIIa) and (IIIb) (Figs. 4 ▸ and 7 ▸), but it is inter­esting to note that the com­ponents of the chains formed by N—H⋯O hydrogen bonds are related by a *c*-glide plane in (IIIa), by a 2_1_ screw axis in (IIIb) and by translation in (IIIc).

**Figure 10:**
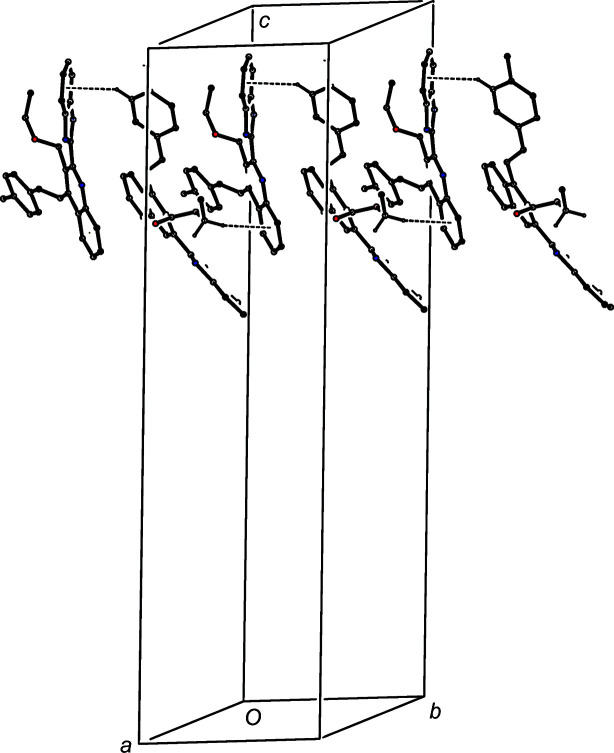
Part of the crystal structure of com­pound (IIIc), showing the formation of a chain parallel to [010] built from C—H⋯π hydrogen bonds (drawn as dashed lines). For the sake of clarity, the minor disorder com­ponent and the H atoms bonded to those atoms not involved in the motif shown have been omitted.

The second substructure in (IIIc) is built from two C—H⋯π hydrogen bonds in which mol­ecule 3 acts as a twofold donor and mol­ecule 2 acts as a twofold acceptor. These two inter­actions generate a chain running parallel to the [010] direction (Fig. 10 ▸). The combination of the chains running parallel to [100] and [010] generates a sheet lying parallel to (001) and occupying the domain 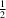 < *z* < 1.0; a second sheet, related to the first by inversion, occupies the domain 0 < *z* < 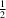 , but there are no direction-specific inter­actions between adjacent sheets.

In summary, therefore, we have developed an efficient and versatile synthetic route to novel hybrid (*E*)-2-(1*H*-benzo[*d*]imidazol-2-yl)-4-styryl­quinolines from very simple starting materials; we have fully characterized by spectroscopic means (IR, ^1^H and ^13^C NMR spectroscopy, and HR-MS) three representative examples, together with one inter­mediate on the pathway to each product, and we have determined the mol­ecular and supra­molecular structures of the three products thus formed.

## Supplementary Material

Crystal structure: contains datablock(s) global, IIIa, IIIb, IIIc. DOI: 10.1107/S2053229622010063/ky3223sup1.cif

Structure factors: contains datablock(s) IIIa. DOI: 10.1107/S2053229622010063/ky3223IIIasup2.hkl

Structure factors: contains datablock(s) IIIb. DOI: 10.1107/S2053229622010063/ky3223IIIbsup3.hkl

Structure factors: contains datablock(s) IIIc. DOI: 10.1107/S2053229622010063/ky3223IIIcsup4.hkl

Supporting information file. DOI: 10.1107/S2053229622010063/ky3223IIIasup5.cml

Supporting information file. DOI: 10.1107/S2053229622010063/ky3223IIIbsup6.cml

Supporting information file. DOI: 10.1107/S2053229622010063/ky3223IIIcsup7.cml

CCDC references: 2213571, 2213572, 2213573

## Figures and Tables

**Figure 2:**
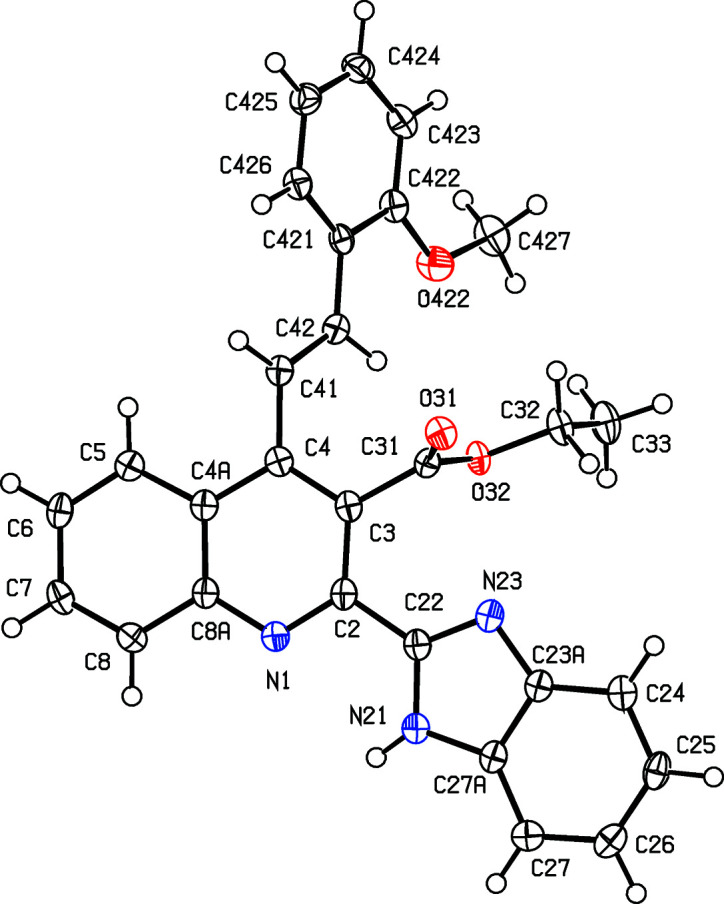
The mol­ecular structure of com­pound (IIIb), showing the atom-labelling scheme. Displacement ellipsoids are drawn at the 50% probability level.

**Table 1:** Experimental details Experiments were carried out at 100 K with Mo *K*α radiation using a Bruker D8 Venture diffractometer. Absorption was corrected for by multi-scan methods (*SADABS*; Bruker, 2016 ▸). H atoms were treated by a mixture of independent and constrained refinement.

	(IIIa)	(IIIb)	(IIIc)
Crystal data			
Chemical formula	C_27_H_20_ClN_3_O_2_	C_28_H_23_N_3_O_3_	C_28_H_23_N_3_O_2_(+solvent)
*M*_r_	453.91	449.49	433.49
Crystal system, space group	Monoclinic, *P*2_1_/*c*	Monoclinic, *P*2_1_/*n*	Monoclinic, *P*2_1_/*n*
*a*, *b*, *c* (Å)	12.1791 (5), 18.8348 (7), 10.7533 (4)	15.8086 (14), 6.9536 (6), 20.4101 (19)	20.2611 (7), 9.8675 (4), 36.8434 (14)
β (°)	114.242 (1)	94.330 (4)	104.332 (1)
*V* (Å^3^)	2249.19 (15)	2237.2 (3)	7136.7 (5)
*Z*	4	4	12
μ (mm^−1^)	0.20	0.09	0.08
Crystal size (mm)	0.18 × 0.16 × 0.12	0.16 × 0.12 × 0.08	0.18 × 0.12 × 0.06

Data collection			
*T*_min_, *T*_max_	0.928, 0.976	0.817, 0.993	0.917, 0.995
No. of measured, independent and observed [*I* > 2σ(*I*)] reflections	56016, 4969, 4417	41777, 5131, 3453	151790, 17718, 10436
*R*_int_	0.051	0.132	0.188
(sin θ/λ)_max_ (Å^−1^)	0.642	0.650	0.667

Refinement			
*R*[*F* ^2^ > 2σ(*F* ^2^)], *wR*(*F* ^2^), *S*	0.037, 0.096, 1.05	0.059, 0.133, 1.06	0.096, 0.216, 1.08
No. of reflections	4969	5131	17718
No. of parameters	302	312	921
No. of restraints	0	0	7
Δρ_max_, Δρ_min_ (e Å^−3^)	0.61, −0.32	0.25, −0.26	0.71, −0.41

Computer programs: *APEX3* (Bruker, 2018 ▸), *SAINT* (Bruker, 2017 ▸), *SHELXT2014* (Sheldrick, 2015*a* ▸), *SHELXL2014* (Sheldrick, 2015*b* ▸) and *PLATON* (Spek, 2020 ▸).

**Table 2:** Selected torsion and dihedral angles (°) for com­pounds (IIIa)–(IIIc) The term dihedral here refers to the dihedral angle between the pyridine and the imidazole rings. In order to specify an asymmetric unit in which the three independent mol­ecules of (IIIc) were linked by hydrogen bonds, it was necessary to select mol­ecule 2 (*x* = 2) to be the conformational enanti­omer opposite from those selected for mol­ecules 1 and 3 (*x* = 1 and 3) (see text). For ease of com­parison, the values of the torsion angles cited for *x* = 2 refer to the inverted mol­ecule at (−*x*, −*y*, −*z*) so that the values refer to corresponding conformational enanti­omers for all three mol­ecules, with positive values for the torsion angles C*x*3—C*x*4—C*x*41—C*x*41.

**Compounds (IIIa) and (IIIb)**			
Parameter	(IIIa)	(IIIb)	
N1—C2—C22—N21	10.95 (18)	8.2 (2)	
C2—C3—C31—O31	−99.75 (18)	−98.1 (3)	
C2—C3—C31—O32	81.29 (15)	85.2 (2)	
C3—C4—C41—C42	53.3 (2)	49.8 (3)	
C41—C42—C421—C422	169.18 (14)	−167.1 (2)	
Dihedral	11.62 (1)	8.52 (3)	
			
**Compound (IIIc)**			
Parameter	*x* = 1	*x* = 2	*x* = 3
N*x*1—C*x*2—C*x*22—N*x*21	9.0 (5)	31.1 (4)	22.1 (4)
C*x*2—C*x*3—C*x*31—O*x*31	84.4 (5)	−103.2 (4)	−102.2 (4)
C*x*2—C*x*3—C*x*31—O*x*32	−97.2 (4)	78.9 (4)	78.3 (4)
C*x*3—C*x*4—C*x*41—C*x*42	65.6 (6)	42.9 (5)	47.0 (5)
C*x*41—C*x*42—C*x*43—C*x*44	−168.5 (4)	155.1 (4)	171.9 (4)
Dihedral	12.4 (3)	32.26 (11)	23.24 (18)

**Table 3:** Hydrogen bonds (Å, °) for com­pounds (IIIa)–(IIIc) *Cg*1–*Cg*5 represent the centroids of the rings C23*A*/C24–C27/C27*A*, N1/C2–C4/C4*A*/C8*A*, C421–C426, C24*A*/C25–C28/C28*A* and C23*A*/C27*A*/C227/C226/C225/C224, respectively.

	*D*—H⋯*A*		*D*—H	H⋯*A*	*D*⋯*A*	*D*—H⋯*A*
(IIIa)	N21—H21⋯O31^i^		0.832 (19)	2.379 (18)	3.0764 (16)	141.8 (16)
	C422—H422⋯*Cg*1^ii^		0.95	2.53	3.4420 (17)	168
	C426—H426⋯*Cg*2^iii^		0.95	2.68	3.5161 (18)	148
(IIIb)	N21—H21⋯O31^iv^		0.86 (3)	2.45 (2)	3.081 (3)	130.4 (19)
	C7—H7⋯*Cg*3^iii^		0.95	2.95	3.704 (2)	138
	C33—H33*B*⋯*Cg*3^v^		0.98	2.98	3.768 (3)	139
(IIIc)	N121—H121⋯O231		0.78 (4)	2.17 (4)	2.876 (4)	150 (4)
	N221—H221⋯O331		0.83 (4)	2.13 (4)	2.882 (4)	151 (4)
	N321—H321⋯O131^vi^		0.84 (4)	2.20 (4)	2.910 (4)	141 (3)
	N321—H321⋯O431^vi^		0.84 (4)	2.35 (5)	4.03 (3)	137 (3)
	C127—H127⋯N223		0.95	2.58	3.432 (5)	149
	C227—H227⋯N323		0.95	2.62	3.522 (5)	159
	C332—H32*B*⋯*Cg*4^iv^		0.99	2.80	3.702 (5)	152
	C347—H347⋯*Cg*5^vii^		0.95	2.70	3.548 (5)	149

Symmetry codes: (i) *x*, −y + 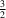 , *z* + 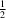 ; (ii) −*x* + 2, y − 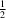 , −*z* + 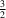 ; (iii) −*x* + 1, −y + 1, −*z* + 1; (iv) −*x* + 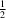 , y + 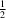 , −*z* + 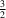 ; (v) −*x*, −y + 1, −*z* + 1; (vi) *x* − 1, y, *z*; (vii) −*x* + 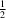 , y − 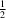 , −*z* + 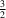 .

## References

[bb1] Afzal, O., Kumar, S., Haider, M. R., Ali, M. R., Kumar, R., Jaggi, M. & Bawa, S. (2015). *Eur. J. Med. Chem.* **97**, 871–910. 10.1016/j.ejmech.2014.07.044 25073919

[bb2] Agrawal, R., Jain, P. & Dikshit, S. N. (2012). *Curr. Drug Targets*, **13**, 863–875. 10.2174/138945012800564059 22250655

[bb3] Bellam, M., Gundluru, M., Sarva, S., Chadive, S., Netala, V. R., Tartte, V. & Cirandur, S. R. (2017). *Chem. Heterocycl. C*, **53**, 173–178.

[bb4] Bernstein, J., Davis, R. E., Shimoni, L. & Chang, N.-L. (1995). *Angew. Chem. Int. Ed. Engl.* **34**, 1555–1573.

[bb5] Bruker (2016). *SADABS*. Bruker AXS Inc., Madison, Wisconsin, USA.

[bb6] Bruker (2017). *SAINT*. Bruker AXS Inc., Madison, Wisconsin, USA.

[bb7] Bruker (2018). *APEX3*. Bruker AXS Inc., Madison, Wisconsin, USA.

[bb8] Cosimelli, B., Taliani, S., Greco, G., Novellino, E., Sala, A., Severi, E., Da Settimo, F., La Motta, C., Pugliesi, I., Antonioli, L., Fornai, M., Colucci, R., Blandizzi, C., Daniele, S., Trincavelli, M. L. & Martini, C. (2011). *ChemMedChem*, **6**, 1909–1918. 10.1002/cmdc.201100284 21796795

[bb9] Etter, M. C. (1990). *Acc. Chem. Res.* **23**, 120–126.

[bb10] Etter, M. C., MacDonald, J. C. & Bernstein, J. (1990). *Acta Cryst.* B**46**, 256–262. 10.1107/s0108768189012929 2344397

[bb11] Ferguson, G., Glidewell, C., Gregson, R. M. & Meehan, P. R. (1998*a*). *Acta Cryst.* B**54**, 129–138.

[bb12] Ferguson, G., Glidewell, C., Gregson, R. M. & Meehan, P. R. (1998*b*). *Acta Cryst.* B**54**, 139–150.

[bb13] Flack, H. D. & Bernardinelli, G. (1999). *Acta Cryst.* A**55**, 908–915. 10.1107/s0108767399004262 10927300

[bb14] Gregson, R. M., Glidewell, C., Ferguson, G. & Lough, A. J. (2000). *Acta Cryst.* B**56**, 39–57. 10.1107/s0108768199006072 10735443

[bb15] Groom, C. R., Bruno, I. J., Lightfoot, M. P. & Ward, S. C. (2016). *Acta Cryst.* B**72**, 171–179. 10.1107/S2052520616003954 PMC4822653 27048719

[bb16] Gurvinder, S., Maninderjit, K. & Mohan, C. (2013). *Int. Res. J. Pharm.* **4**, 82–87.

[bb17] Hernández-Romero, D., Rosete-Luna, S., López-Monteon, A., Chávez-Piña, A., Pérez-Hernández, N., Marroquín-Flores, J., Cruz-Navarro, A., Pesado-Gómez, G., Morales-Morales, D. & Colorado-Peralta, R. (2021). *Coord. Chem. Rev.* **439**, 213930.

[bb18] Hranjec, M., Pavlović, G., Marjanović, M., Kralj, M. & Karminski-Zamola, G. (2010). *Eur. J. Med. Chem.* **45**, 2405–2417. 10.1016/j.ejmech.2010.02.022 20207049

[bb19] Hu, Y.-Q., Gao, C., Zhang, S., Xu, L., Xu, Z., Feng, L.-S., Wu, X. & Zhao, F. (2017). *Eur. J. Med. Chem.* **139**, 22–47. 10.1016/j.ejmech.2017.07.061 28800458

[bb20] Jagdale, D. & Patil, P. (2019). *World J. Pharm. Pharm. Sci.* **8**, 311–328.

[bb21] Kaur, K., Jain, M., Reddy, R. P. & Jain, R. (2010). *Eur. J. Med. Chem.* **45**, 3245–3264. 10.1016/j.ejmech.2010.04.011 20466465

[bb22] Kidwai, M., Jahan, A. & Bhatnagar, D. (2010). *J. Chem. Sci.* **122**, 607–612.

[bb23] Lauria, A., La Monica, G., Bono, A. & Martorana, A. (2021). *Eur. J. Med. Chem.* **220**, 113555. 10.1016/j.ejmech.2021.113555 34052677

[bb24] Lin, S. & Yang, L. (2005). *Tetrahedron Lett.* **46**, 4315–4319.

[bb25] Mantu, D., Antoci, V., Moldoveanu, C., Zbancioc, G. & Mangalagiu, I. I. (2016). *J. Enzyme Inhib. Med. Chem.* **31**, 96–103. 10.1080/14756366.2016.1190711 27250919

[bb26] Matada, B. S., Pattanashettar, R. & Yernale, N. G. (2021). *Bioorg. Med. Chem.* **32**, 115973. 10.1016/j.bmc.2021.116098 33740641

[bb27] Meléndez, A., Plata, E., Rodríguez, D., Ardila, D., Guerrero, S. A., Acosta, L. M., Cobo, J., Nogueras, M. & Palma, A. (2020). *Synthesis*, **52**, 1804–1822.

[bb28] Moss, G. P. (1996). *Pure Appl. Chem.* **68**, 2193–2222.

[bb29] Musiol, R. (2017). *Exp. Opin. Drug. Discov.* **12**, 583–597. 10.1080/17460441.2017.1319357 28399679

[bb30] Nayak, A. (2004). *Expert Opin. Pharmacother.* **5**, 679–686. 10.1517/14656566.5.3.679 15013935

[bb31] Orozco, D., Kouznetsov, V. V., Bermúdez, A., Vargas Méndez, L. Y., Mendoza Salgado, A. R. & Meléndez Gómez, C. M. (2020). *RSC Adv.* **10**, 4876–4898. 10.1039/c9ra09905k PMC9049580 35498276

[bb32] Perin, N., Nhili, R., Cindrić, M., Bertoša, B., Vušak, D., Martin-Kleiner, I., Laine, W., Karminski-Zamola, G., Kralj, M., David-Cordonnier, M. H. & Hranjec, M. (2016). *Eur. J. Med. Chem.* **122**, 530–545. 10.1016/j.ejmech.2016.07.007 27448912

[bb33] Renhowe, P. A., Pecchi, S., Shafer, C. M., Machajewski, T. D., Jazan, E. M., Taylor, C., Antonios-McCrea, W., McBride, C. M., Frazier, K., Wiesmann, M., Lapointe, G. R., Feucht, P. H., Warne, R. L., Heise, C. C., Menezes, D., Aardalen, K., Ye, H., He, M., Le, V., Vora, J., Jansen, J. M., Wernette-Hammond, M. E. & Harris, A. L. (2009). *J. Med. Chem.* **52**, 278–292. 10.1021/jm800790t 19113866

[bb34] Riddell, F. G. & Rogerson, M. (1996). *J. Chem. Soc. Perkin Trans. 2*, pp. 493–504.

[bb35] Riddell, F. G. & Rogerson, M. (1997). *J. Chem. Soc. Perkin Trans. 2*, pp. 249–256.

[bb36] Rodríguez, D., Guerrero, S. A., Palma, A., Cobo, J. & Glidewell, C. (2020). *Acta Cryst.* C**76**, 883–890. 10.1107/S2053229620010803 PMC7474186 32887859

[bb37] Salahuddin, Shaharyar, M. & Mazumder, A. (2017). *Arab. J. Chem.* **10**, S157–S173.

[bb38] Sheldrick, G. M. (2015*a*). *Acta Cryst.* A**71**, 3–8.

[bb39] Sheldrick, G. M. (2015*b*). *Acta Cryst.* C**71**, 3–8.

[bb40] Singh, M. P., Sasmal, S., Lu, W. & Chatterjee, M. N. (2000). *Synthesis*, **2000**, 1380–1390.

[bb41] Singhal, S., Khanna, P., Panda, S. S. & Khanna, L. (2019). *J. Heterocycl. Chem.* **56**, 2702–2729.

[bb42] Spek, A. L. (2015). *Acta Cryst.* C**71**, 9–18. 10.1107/S2053229614024929 25567569

[bb43] Spek, A. L. (2020). *Acta Cryst.* E**76**, 1–11. 10.1107/S2056989019016244 PMC6944088 31921444

[bb45] Vera, D. R., Mantilla, J. P., Palma, A., Cobo, J. & Glidewell, C. (2022). *Acta Cryst.* C**78**, 524–530. 10.1107/S2053229622008634 PMC9533309 36196785

[bb46] Yadav, P. & Shah, K. (2021). *Bioorg. Chem.* **109**, 104639. 10.1016/j.bioorg.2021.104639 33618829

[bb47] Yaragorla, S. & Vijaya Babu, P. (2017). *Tetrahedron Lett.* **58**, 1879–1882.

